# Planning Socially Expressive Mobile Robot Trajectories

**DOI:** 10.3390/s24113533

**Published:** 2024-05-30

**Authors:** Philip Scales, Olivier Aycard, Véronique Aubergé

**Affiliations:** 1 GIPSA-Laboratory, University Grenoble Alpes, CNRS, Grenoble INP, 38000 Grenoble, France; 2 LIG-Laboratory, University Grenoble Alpes, CNRS, Grenoble INP, 38000 Grenoble, France

**Keywords:** trajectory planning, human–robot interaction, perception experiment, logistic regression

## Abstract

Many mobile robotics applications require robots to navigate around humans who may interpret the robot’s motion in terms of social attitudes and intentions. It is essential to understand which aspects of the robot’s motion are related to such perceptions so that we may design appropriate navigation algorithms. Current works in social navigation tend to strive towards a single ideal style of motion defined with respect to concepts such as comfort, naturalness, or legibility. These algorithms cannot be configured to alter trajectory features to control the social interpretations made by humans. In this work, we firstly present logistic regression models based on perception experiments linking human perceptions to a corpus of linear velocity profiles, establishing that various trajectory features impact human social perception of the robot. Secondly, we formulate a trajectory planning problem in the form of a constrained optimization, using novel constraints that can be selectively applied to shape the trajectory such that it generates the desired social perception. We demonstrate the ability of the proposed algorithm to accurately change each of the features of the generated trajectories based on the selected constraints, enabling subtle variations in the robot’s motion to be consistently applied. By controlling the trajectories to induce different social perceptions, we provide a tool to better tailor the robot’s actions to its role and deployment context to enhance acceptability.

## 1. Introduction

There is an increasing number of application domains for mobile robots operating in environments alongside humans, both in public spaces (e.g., train stations and shops) as well as spaces such as hospitals [1], care-homes, or private homes [2]. In the early days of human–robot interaction research, it was quickly established that using traditional navigation algorithms that only consider humans as obstacles results in unacceptable robot behaviour. This led to the emergence of the field of Social Navigation (SN) [3], which aims to design human-aware navigation algorithms to improve acceptability. The deployment of mobile robots in human environments requires overcoming both technical challenges as well as human factors and social perception challenges. An important example of such perception challenges is the Uncanny Valley phenomenon [4], whereby the appearance of a robot greatly influences its likability and human affinity with it, which will inevitably impact robot adoption and integration into society. Another major dimension of the Uncanny Valley is the role of movement dynamics in changing human perception of the robot [5] as well as the role of the robot’s attitude [6], although these dimensions have not received as much attention in studies. Therefore, it is necessary to determine whether a robot’s movements may impact human social perception of the robot’s attitudes in order to improve acceptability and interaction quality.

Recent works in robotics have been carried out on the topic of functional expressive motion generation [7], whereby subtle aspects of a robot’s movement can be modified to express the robot’s intentions or emotions while simultaneously executing a task. In [8], it was shown that a humanoid robot arm handing over an object with a rude or gentle attitude could influence the human interacting with the robot. While algorithms have been proposed to generate legged robot movements expressing emotions such as happy or sad [9], navigation algorithms for mobile robots focus on other dimensions such as naturalness and comfort [10,11,12,13]. These dimensions are different from social attitudes such as aggressiveness, hesitancy, or politeness, whose impact on human interactions has been studied, particularly in the field of vocal prosody [14,15]. It is unknown which movement features may lead to different perceptions of social attitudes in mobile robots, and existing navigation algorithms are not able to adjust the robot’s motion so that it generates different perceptions of social attitudes. This lack of understanding of the social perception of robot motion, and the inability to adjust navigation features, may be partly responsible for the low acceptance that can be observed when mobile robots are deployed in real environments [16,17].

In this paper, we explore two questions in order to improve acceptability and integration of robots in human environments. Firstly, which features of a mobile robot’s motion may lead a person to interpret its motion in terms of social attitudes such as aggressive, gentle, authoritative, or polite? Secondly, how may we formalize these features and incorporate them into a novel navigation algorithm capable of altering the navigation style to adjust how the robot is perceived by humans? In order to answer these questions, we make the following contributions:

- We build a statistical model of human perception of different combinations of trajectory features capturing a robot’s movement dynamics, bringing new knowledge on human social perception of robot motion.
- We formalize these trajectory features found to cause different social perceptions and design a novel optimization-based trajectory planning algorithm that can accurately reproduce the social motion features while performing a navigation task.

Our statistical analysis demonstrates that subtle motion features can strongly shape perception of the attitudes and physical attributes of mobile robots. Our algorithm enables control over the robot’s expression of social attitudes through its motion, which was not possible using existing algorithms focused on comfort and naturalness or on the expression of emotions.

The structure of the paper is as follows: Section 2 reviews existing approaches for evaluating and designing social navigation algorithms. Section 3 summarizes the perception experiment from our prior work and presents our approach to model the relationship between the robot’s subtle motion features and the participant’s perception of social attitudes and physical characteristics. Section 4 describes our trajectory planning algorithm formulated as a constrained optimization problem using novel constraints and control input formulations, which ensure that the robot’s motion always contains the features relevant for altering human perception of the robot. Section 5 presents the implementation and validation of our algorithm on a real mobile robot, demonstrating that the plans can be accurately reproduced by the robot in a way that maintains the key movement features. Finally, we draw conclusions and present ideas for future work in Section 6.

## 2. State of the Art

### 2.1. Design and Evaluation of Social Navigation Algorithms

Works in the field of Social Navigation [18] typically concern themselves with enabling mobile robots to navigate in complex environments [19], around many (potentially dynamic) pedestrians [20], and modeling uncertainty of surrounding pedestrian motion [21]. Social navigation approaches tend to focus on ensuring the robot plans safe, comfortable, and natural motion, following the definitions in [3]. Questionnaires such as the Godspeed Questionnaire Series [22], Negative Attitudes towards Robots Scale (NARS) [23], Perceived Social Intelligence scale (PSI) [24], or Robot Social Attributes Scale (RoSAS) [25] are often used to assess naturalness, comfort, and likability, aiming to maximize all of them with a single navigation style. Although these metrics are useful, there are still issues with the acceptability of mobile robots, particularly when humans attribute social intentions or attitudes to a mobile robot’s navigation. Vocal prosody (pitch, rhythm, and tone) has been widely studied as a means to convey social attitudes and relation information in interactions between both humans and robots [26,27]. In addition to studying vocal prosody, ref. [28] observed that the changes in a participant’s vocal prosody over the course of an interaction with a robot were aligned with changes in their spatial behavior, gaze, and voice quality [29]. We hypothesize that a robot’s navigation movement prosody (i.e., subtle differences in it’s style of navigation [30]) may play a role in how people perceive different social attitudes. This is different to existing works that study naturalness and comfort [10] or the expression of emotions or intent through motion [7].

A typical approach to design a social navigation algorithm is to first develop the algorithm, then subsequently evaluate the impact of the motions it generates. When designing the algorithm, the social aspects can either be implicitly incorporated through machine-learning methods that aim to replicate average human navigation [31,32] or explicitly by manually observing and modeling human behavior [33]. Another approach is to implement existing models of human behavior such as the Social Force Model [34]. Spatial and proximity factors are the most commonly addressed in earlier works [35], often being derived from the concept of proxemics [36]. The algorithm is then used to control a real or simulated robot in order to conduct experiments to evaluate the algorithm. Participants can be asked to compare different navigation algorithms [37,38] or the same algorithm with different parameters such as avoidance distance or speed [39]. Although these approaches enable researchers to evaluate the whole algorithm in terms of its efficiency, comfort, or naturalness, they do not allow a precise understanding of exactly which features of the generated motions are responsible for each aspect of the evaluation.

We propose to first assess which motion variables are important by using hand-crafted motions built through a systematic combination of different motion features across several motion variables. To propose the set of variables to be studied, we make analogies to the variables known to impact voice prosody dynamics, which are known to impact social interaction [15,28,29,40]. The selection and range of the variables reflect our robot’s mechanical constraints and capabilities. Using systematically designed motions aids the understanding of the dimensions at play by using them in perception experiments. Among the initially proposed variables, only those that are found to have an impact on the person’s perception of the robot will be kept, and these will be used to guide the design of our social navigation algorithm.

### 2.2. Algorithmic Approaches for Social Navigation

The goal of social navigation and expressive navigation works is to generate motion that accomplishes a physical task while taking into account a variety of metrics that account for human presence in the environment, as opposed to traditional navigation, which aims to minimize time or path length. This is reflected by recent works from social navigation and functional expressive motion generation turning towards similar methods by encoding the desired trajectory features into cost functions and/or constraints, followed by a search or optimization algorithm to generate trajectories that best match the social or expressive features. These objectives are often in conflict with each other. Formulating trajectory generation as an optimization problem is a common approach both within social robotics [41,42] and in other areas such as crowd behavior generation [43]. A typical solution when faced with conflicting objectives is to adopt a scalarization approach to treat the multi-objective problem as a single-objective problem, usually by computing a weighted average of cost terms [18]. This requires tuning the weights to obtain the desired behavior, balancing the different social and expressive objectives as well as the task objectives such as making progress towards a navigation goal.

In [44], the authors propose two costs, modeling visibility of the robot by the human and a proxemics-inspired personal space. They propose either a weighted average or taking the maximum out of the two costs. This choice depends on the task and balance between criteria, and the weights should be tuned according to the properties of the task. In a more recent work, ref. [41] present an approach that jointly plans cooperative trajectories for a single human and the robot, accounting for metrics such as the expected time to collide with the person, modulating robot velocity when near the person, and legibility of the trajectory. Other aspects have been modeled such as preferring deceleration rather than changing path shape to negotiate crossing a person [45], maintaining a desired position and velocity while accompanying a person [46], and avoiding intrusion into group formations and the information processing space in front of people [47]. Similarly, for expressive motion, ref. [48] uses a weighted sum of costs; however, they also explore the use of learned weights based on participant perception of emotion in the robot arm’s trajectories, avoiding the manual tuning process. Some features are shared across several works, with the most common being personal space around people derived from proxemics [35]; however, most works consist precisely of proposing their own novel cost or constraint, leading to each work using different subsets of cost terms.

While trade-offs between traditional task performance metrics and social or expressive features are inevitable, the issue with these approaches is that there is limited control over how the trade-off is performed. In some works, this trade-off is enforced more explicitly, such as in [49], where the expressive features for a robot arm can only be expressed through degrees of freedom that have absolutely no effect on the practical task. On the contrary, in [50], the authors first develop a smooth parameterized control law for their autonomous wheelchair such that it produces graceful motion. Their trajectory planner optimizes over the parameter space defined by the control law, thus enforcing a given style of motion, regardless of the impact on task performance.

Formulating the problem as a trajectory optimization provides a general framework consisting of cost functions and constraints that can be combined to model complex navigation styles. Machine-learning techniques are also popular in social navigation [31,32,51]; however, they require many demonstrations or large annotated datasets, which would have to be annotated with the corresponding social perceptions. Currently, such datasets with annotations of human social perception of the mobile robot do not exist and would be complex and time-consuming to create. Furthermore, learned navigation models lack the explainability and controllability given by optimization-based approaches. Hence, we adopt a trajectory optimization problem formulation. It is crucial that our algorithm generates motions that are very accurately matched to those we use to construct our model of human perception of robot motion. For this reason, rather than modeling the desired motion through the cost function, which would make the desired prosody features subject to trade-offs with other cost terms modeling the functional task to be achieved, we propose to design specific prosody constraints to enforce the desired properties of motion. In this sense, our approach is inspired by [50], since we also restrict the valid trajectory space a priori according to the desired style of motion. The constraints must take into account the consistency of the robot’s motion style over time as well as its ability to plan a future trajectory with appropriate movement prosody.

## 3. Model of Human Social Perception of Mobile Robot Motion

In this section, we discuss our method for modeling how different variations in a mobile robot’s motion and appearance influence people’s social and physical perception of the robot. In our prior work [30], we designed a robot motion corpus that consists of motion and appearance variables, which are combined to define many different styles of motion. The corpus was used to conduct perception experiments by asking participants to rate their perception of a mobile robot by viewing videos of it performing motions from the corpus. In the prior work, early experimental results using simple chi-square tests suggested that the variables included in our corpus had significant impacts on people’s perception of the robot. In this paper, we present experimental results from a larger participant pool as well as the results of mixed effects logistic regression modeling to determine precisely how each corpus variable impacts the probability of different social perceptions of the robot. In the following subsections, we first give the definitions of each of the corpus variables, followed by the ten perceptual scales we used to evaluate participant perceptions; lastly, we present the results of the regression analyses.

### 3.1. Robot Motion Corpus Background

In this subsection, we briefly present our motion corpus and describe which features of the robot are manipulated. Firstly, we present the motion variables that impact the robot’s motion by defining its velocity, acceleration, and style of motion. Secondly, we present the appearance variables that also contribute to changing a person’s perceptual experience when interacting with a mobile robot. The corpus is available online at the following address: https://osf.io/5csrg/ (accessed on 25 March 2024), and an example video is available at: https://youtu.be/EiH8o1PjlOw (accessed on 25 March 2024).

#### 3.1.1. Motion Variables

The goal of the motion corpus variables is to uniquely define a velocity profile for the robot’s linear (forward translation) velocity. Each variable affects different features of the profile determining the robot’s velocity over time in order to perform a point-to-point straight line motion. The most basic way to achieve such a motion would be to perform acceleration using the maximal motor acceleration followed by a similar deceleration, forming a triangular velocity profile. The first variable is the kinematics type, which defines both the maximum velocity as well as the slope of the profile, i.e., the acceleration value. This variable has three values (low, medium, and high), enabling comparisons between the different values listed in Table 1.

**Figure 1:**
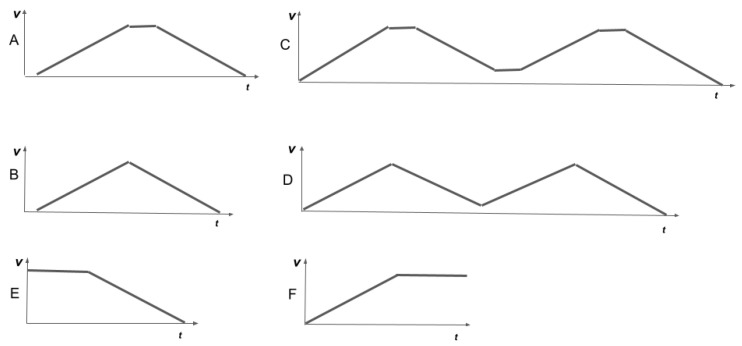
Illustrations of the effect of the six motion sequence values on the velocity profiles, shown in the six subfigures (**A**–**F**). The slope and maximum values of the profiles are determined by the kinematics variable. These profiles use the smooth variant.

The next motion variable is the motion sequence. This variable affects the overall shape of the velocity profile by defining the ordering and succession of acceleration and deceleration phases, shown in Figure 1. The previous example of a single acceleration followed by a deceleration corresponds to motion sequence B. We introduce two features that impose variations of this sequence: pauses and hesitations. A pause motion sequence is defined as always introducing a short, constant velocity phase between an acceleration and deceleration phase, transforming the profile from a triangular shape to a trapezoidal shape, as in sequence A. A hesitation motion sequence is defined as introducing a “V” shape into the profile after an acceleration phase, meaning that the robot decelerates partially before accelerating back up to its previous velocity peak, as in sequence D. Pauses and hesitations are combined in sequence C. Sequences E and F are simply extensions of profile B, representing only the start or end of a robot’s motion as it arrives or leaves.

**Figure 2:**
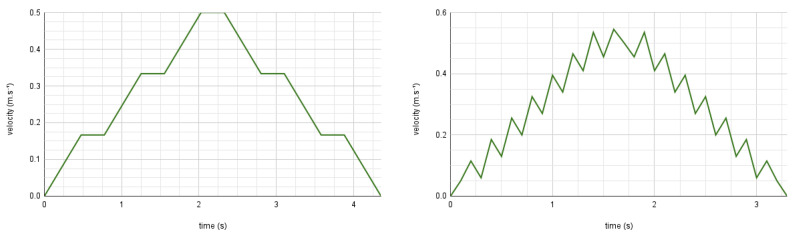
Velocity profiles resulting from combining the increment (**left**) or saccade (**right**) variants with motion sequence A and medium kinematics.

The last motion variable is the variant. The previous illustrations represent profiles using the smooth variant, where each acceleration and deceleration is a linear segment. In contrast, we define two other variants that alter the overall style of the robot’s motion, shown in Figure 2. The saccade variant is defined as the velocity profile oscillating periodically to induce stuttering and shaking into the robot’s motion. The increment variant is defined as dividing the acceleration and deceleration phases into three separate phases interleaved by short constant velocity phases, creating a stepping or incremental motion.

#### 3.1.2. Appearance Variables

In addition to altering the robot’s velocity profile, we use several variables to alter the visual appearance of the robot. The first variable is the robot base type, which is either stable or unstable. When the base is unstable, the robot’s front and back balancing wheels are loosened making the robot tilt backwards or forwards when accelerating or decelerating. The robot’s head is also loosened, making it shake and move when using the shaking motion induced by the saccade variant.

The second appearance variable is the head motion. The robot’s head can be fixed in different orientations: either straight ahead or 90° to the side. Two other settings involve the head rotating from the straight to the side orientation or from the side to the straight orientation while the robot performs its motion. In the videos presented to the participants, the side orientation directs the robot’s gaze towards the viewer, whereas the straight orientation directs the gaze towards the direction of travel of the robot—to the right-hand side of the video frame.

The third appearance variable is the eye shape. The LED eye displays on the robot’s head are set to three different display settings. The first setting corresponds to white round eyes, which represent a neutral appearance. The second setting corresponds to green squinting eyes that represent a colder, unsettling appearance. In general, a robot’s appearance has been found to impact interaction in prior studies [52,53]; hence, we include these variables to be able to distinguish the effect of the appearance from the effect of the motion variables.

### 3.2. Perceptual Scales

In Table 2, we present the ten semantic differential scales used in order to gather participants’ social and physical impressions of the robot. Part of the scales represent attitudes towards others, such as Authoritative–Polite, Aggressive–Gentle, Inspires–Doesn’t inspire confidence, Nice–Disagreeable, and Tender–Insensitive. Evaluating these perceptions involves a directed attitude. Confident–Hesitant is more related to the robot’s own affective state. The remaining scales capture physical perceptions of the robot, with Sturdy–Frail, Strong–Weak, Smooth–Abrupt, and Rigid–Supple. The scales were chosen based on words that participants in prior HRI studies had used to self-annotate their own recorded interaction data after a long experiment with a small butler robot [26,27]. For more details about the choice of the adjectives in our scales, we refer the reader to [30].

### 3.3. Logistic Regression Modeling

#### 3.3.1. Method

During the experiment, each participant rated 45 different videos each showing a unique combination of the corpus variable values along the 10 binary perceptual scales. A total of n=100 participants of various ages (M = 33.61, SD = 14.76) and genders (56 female, 36 male, 6 other, and 2 without response) were recruited through social media, local university and lab experiment mailing lists, as well as fliers handed out in public spaces. There was no selection criteria other than fluently speaking the language used to express the adjectives (in this study, we used French). Participants were instructed to perform the experiment on a device with a large screen and smooth video playback to ensure good perception of the robot motions. Each of the 450 videos was rated 10 times; hence, each participant performs ten binary classifications for each video, where the input is the set of values for each of the corpus motion variables and the output is a binary choice between the two adjectives of each scale. We chose to fit a mixed effects logistic regression model for each scale [54], allowing us to account for dependencies within the data since participants each provide 45 responses. Each of the corpus variables is treated as a categorical fixed effect, and the participant *id* is used as a random effect. The models were implemented in R using the lme4 package [55]. The logistic models give the probability of the participant choosing the second (rightmost) adjectives of the scales in Table 2. In R syntax, the model structure can be given as follows:

(1) $$\begin{array}{c} scale∼\;kinematics+sequence+variant+eyes+base+head+(1|id) \end{array}$$

We did not include interaction terms in the final model, since pairwise interactions resulted in a worse model fit on the test data, as measured by the Area Under Curve (AUC) of the Receiver Operator Characteristic (ROC) curve [56,57] (average 0.798 with interaction terms, 0.805 without interactions).

The log odds scale used for the logistic regression coefficients is practical for fitting the models and computing predictions; however, it is not a very intuitive scale to interpret the results. An alternative way to understand the results of mixed effect logistic regression is to compute the estimated marginal means (EMM) based on the model predictions [58]. These means represent the average of predicted values of the response variable for each level of the corpus variables. Averaging is performed across all levels of all other variables. In order to establish the relative effects of the different levels of the corpus variables on each scale, we construct contrasts that compare each level’s EMM with the average over all levels. We perform the EMM and contrast computation using the emmeans R package [59].

#### 3.3.2. Results

Table 3 presents the marginal effects of each value of each corpus variable on the perceptual scales. The effects are reported as percentage points, indicating the increase or decrease in the probability of participants selecting the second adjective of the scale when that level of a corpus variable is used, compared to the overall mean. For example, using high kinematics is estimated to decrease the probability of gentle being selected over aggressive, or equivalently, increase the chance of people perceiving the robot as aggressive by 28 p.p. percentage points compared to the overall mean. The statistical significance of the difference between the EMM for a given level and the average EMM over all levels was tested using z-tests. A Holm–Bonferroni correction [60] was applied to the *p*-values to adjust for multiple comparisons. The head rotation variable is not represented in the table, given that the only significant effects were on the sturdy–frail scale, with contrasts 5 p.p. and  −5 p.p. for straight and turn straight values (both p<0.05), respectively.

The contrast values range from −28 p.p. (high kinematics effect on aggressive–gentle) to  27 p.p. (sequence D effect on confident–hesitant), with all values in between, including some null contrasts (sequence C effect on aggressive–gentle). All of the motion corpus variables have statistically significant effects on how participants perceived the robot. Many of the corpus variable values alter the average probability of differing perceptions by 10, 20, or even almost 30 percentage points. In addition to gaining an understanding of the direction and magnitude of the influence of the corpus variables on human perception, the mixed effect logistic regression models can be used to perform predictions of combinations of the corpus variable values that may be perceived.

**Figure 3:**
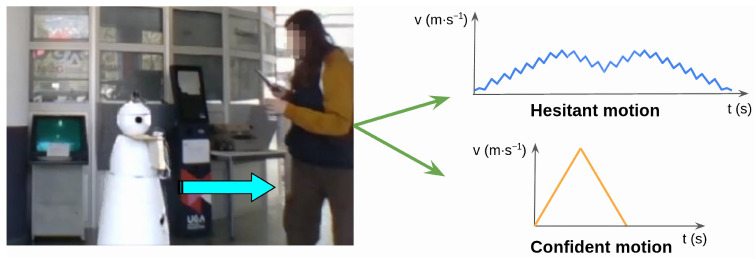
Examples of linear trajectories to approach a person using different velocity, acceleration, and timing features resulting in confident or hesitant perception.

The kinematics and variant variables both have consistently large effects on every perceptual scale, mostly greater than 10 p.p., and greater than 20 p.p. for two to three scales. Kinematics mostly affects the aggressive–gentle and authoritative–polite scales, while variants mostly affect the confident–hesitant and sturdy–frail scales. These are followed by the motion sequence variable with effects greater than 10 p.p. on the confident–hesitant, inspires–doesn’t inspire confidence, and sturdy–frail scales. The base stability and eyes have effects of more than 10 p.p. on two scales. The head variable has little to no effect on any of the scales. These perception experiment results show that all of the corpus variables related to the robot’s linear velocity profiles have strong effects on how the robot is perceived while navigating. These results can be used to derive how to combine the robot motion features in order to generate a desired impression. An example is shown in Figure 3, where the hesitant motion is obtained by combining low kinematics with the saccade variant and motion sequence D; whereas confident motion is obtained with high kinematics, smooth variant, and motion sequence B.

## 4. Algorithm for Trajectory Planning with Configurable Movement Styles

In the previous section, we used logistic regression models to determine how different features of the linear velocity of a mobile robot can impact people’s perception of the robot. The results of the statistical analysis demonstrated which motion features were important, namely, the accelerations, velocities, and inclusion of hesitations and pauses in the movement. In this section, we present our approach to design a trajectory planning algorithm that can be configured in order to enforce these motion features in the generated trajectories. We propose to derive constraints that enable control over the features of the robot’s linear velocity profile to match our model of movement prosody. In this paper, we assume that the environment is static and that there are no obstacles between the robot and the goal in order to focus on accurate reproduction of the movement characteristics from our perception experiment. Firstly, we formalize the velocity profile representation. Secondly, we generalize the profiles to enable trajectories to span different distances. Thirdly, we discuss how a typical trajectory optimization that only constrains motion based on the motor limits cannot allow us to configure the robot’s motion according to our desired movement prosody. Fourthly, we derive a novel set of constraints to enable the trajectory generation to be configured based on the desired prosody. Lastly, we present the algorithm to perform the offline trajectory planning and open loop control to realize the planned motion.

### 4.1. Velocity Profile Representation

In this section, we first introduce the notation that will be used to describe the velocity profiles and then explain how the robot’s motion is controlled when executing the fixed distance velocity profiles from the corpus.

**Figure 4:**
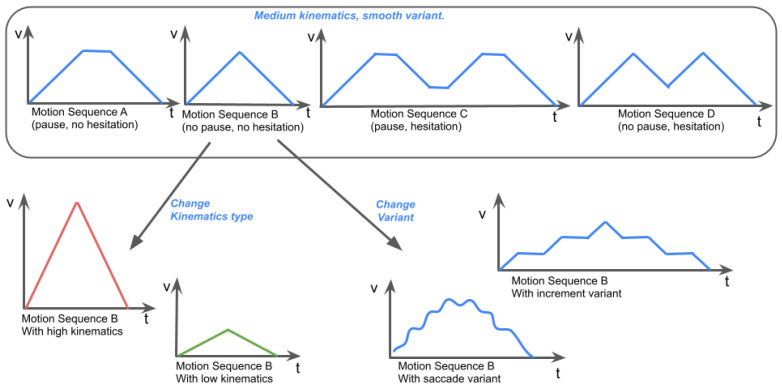
Illustration of the construction of the velocity profiles by combining the motion corpus variables. **Top**: all motion sequences represented with medium kinematics and smooth variant. **Bottom**: profiles resulting from applying different kinematics or variants to motion sequence B. In total, 4×3×3=36 profiles can be obtained by combining the 4 motion sequences with 3 kinematics and 3 variants.

The corpus profiles were constructed by selecting the combination of values for three variables: the motion sequence, kinematics type, and variant. Figure 4 represents how each of these variables alters the shape of the corpus velocity profile.

**Figure 5:**
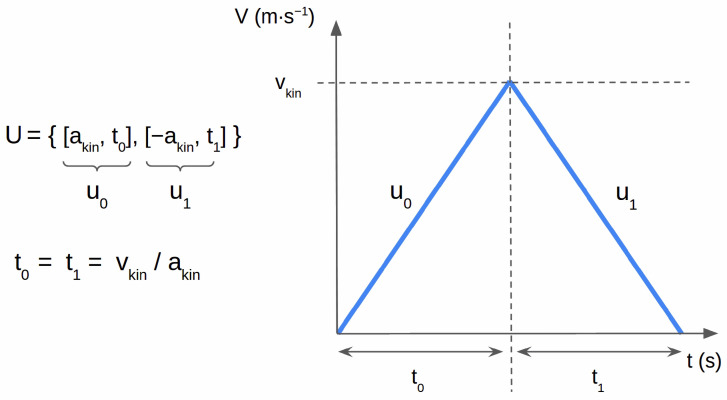
Representation of a corpus velocity profile using motion sequence B (no pauses, no hesitations) and the smooth variant as a sequence *U* of N=2 motion phases $u_{0}$ and $u_{1}$. Values of $v_{kin}$ and $a_{kin}$ depend on the selected kinematics type (medium, low, and high), and dictate the slope and maximum of the velocity profile.

In Figure 5, we give an example of how a corpus velocity profile can be represented as a sequence $U=\{u_{0},u_{1}\ldots u_{N-1}\}$ of *N* motion phases, where $u_{k}=\left[a_{k},t_{k}\right]$. A motion phase $u_{k}$ consists of the slope of the velocity profile (acceleration) $a_{k}$ and a duration $t_{k}$ over which the acceleration is applied. In conjunction with an initial position $x_{0}$ along the robot’s forward axis, and initial linear velocity $v_{0}$, these values define the robot’s trajectory in space and time, and are related through the forward kinematics Equation (2). This equation is simplified with respect to the full differential drive forward kinematics, since we do not control the angular velocity of the robot.

(2) $$\begin{array}{cc} x_{k+1}= & x_{k}+v_{k}t_{k}+\frac{1}{2}a_{k}t_{k}^{2}\\ v_{k+1}= & v_{k}+a_{k}t_{k} \end{array}$$

Since each combination of corpus variables uniquely defines the velocity profile, the distance travelled by the robot for a given type of movement prosody is fixed. For example, the profile using motion sequence B in Figure 5, using medium kinematics ($a_{kin}=a_{medium}=0.35\;m\;\cdot \;s^{-2}$, $v_{kin}=v_{medium}=0.5\;m\;\cdot \;s^{-1}$), results in $t_{0}=t_{1}=\frac{v_{kin}}{a_{kin}}=1.428$ s, meaning the profile makes the robot cover a distance equal to $a_{kin}\times t_{0}^{2}=0.714$ m. In order to change the distance traveled, we need to introduce some degrees of freedom back into the velocity profile. In the following section, we discuss how we add flexibility while maintaining the distinct characteristics of each corpus variable as much as possible.

### 4.2. Generalization of Corpus Profiles to Variable Distances

Executing a given velocity profile results in the robot performing a unique trajectory in space and time with a given length. To design a planning algorithm, we require a formulation where the distance is a free variable. In other words, we want to transform the corpus profiles corresponding to a combination of parameters into a class of profiles that maintains as many characteristics of the original profiles as possible. We keep the piecewise linear curve representation of the corpus profiles, given that using other functions might lead to different impressions. With these limits, changing the distance traveled by following a given velocity profile can be achieved by altering variables of the profile, each of which is already involved in the definition of the corpus profiles:

1. Acceleration and maximum velocity (kinematics type);
2. Successions of accelerations and decelerations (motion sequence and variant);
3. Duration of maximum velocity phase (motion sequence).

**Figure 6:**
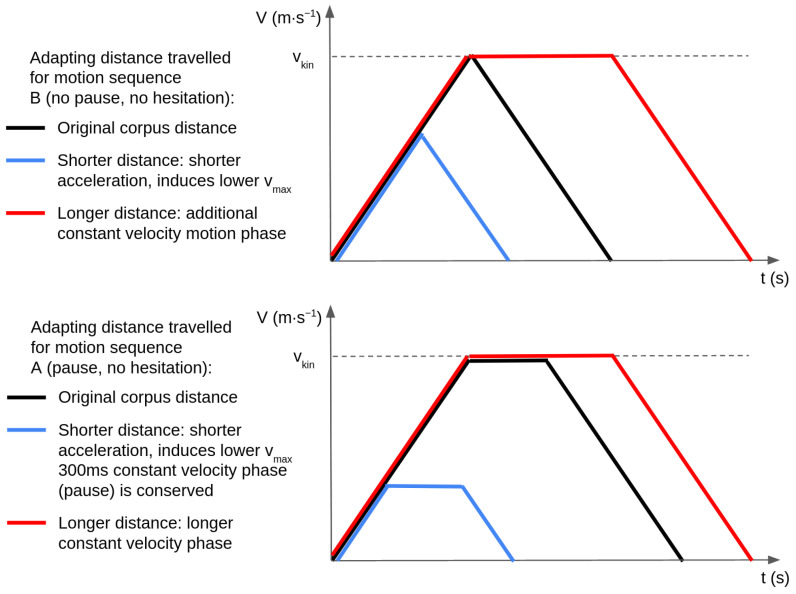
Illustration of the transformation of a corpus velocity profile to travel shorter or longer distances. **Top**: transformation for profiles without pauses or hesitations (sequence B). **Bottom**: transformation for profiles with pauses and without hesitations (sequence A).

Since the kinematics type and variants had high impacts on people’s perceptions of the robot, we choose the third solution of changing the duration of the maximum velocity to adapt the velocity profiles to variable distances. This variable is only partially controlled in our original corpus by the motion sequence. The difference between motion sequences A, C and B, D is the introduction of pauses between acceleration and deceleration phases for sequences A and C, modeled as short (300 ms), constant velocity phases. In order to lengthen profiles, we introduce a constant velocity phase at the maximal velocity. To shorten the profiles, we reduce the maximum velocity while maintaining the profile shape (motion sequences) and the slopes (accelerations of the kinematics type). An example of the transformations applied to alter the distance traveled can be seen in Figure 6. These profiles are achieved by changing the motion phases $u_{k}=\left[a_{k},t_{k}\right]$ by altering the values of the accelerations $a_{k}$ as well as the durations $t_{k}$ such that generating a trajectory to cover a given distance amounts to searching for their optimal values. In the following section, we formalize this trajectory generation process as a constrained optimization problem.

### 4.3. Problem Formulation

We first formalize a typical optimization problem that does not take into account our corpus variables. Moving a robot towards a goal point while accounting for the robot’s mechanical actuation limits can be cast as a discrete-time constrained minimization problem, where we optimize the sequence of control inputs $U=\{u_{0},u_{1}\ldots u_{N-1}\}$ such that the robot minimizes its distance to a goal position $x_{g}$. A control input $u_{k}=\left[a_{k},t_{k}\right]$ corresponds to a motion phase parameterized by a constant acceleration $a_{k}$ and a duration $t_{k}$ over which the acceleration is applied. The durations $t_{k}$ take discrete values, $t_{k}=n*dt$, n∈N, where dt is a constant determining the shortest possible control input duration. The state $x_{k}=\left[x_{k},v_{k}\right]$ of the robot comprises the robot’s position along the *x* axis and its linear velocity *v*, since we focus only on linear motion in this paper. The control $u_{k}$ affects the state $x_{k}$, as described in the kinematics Equation (2).

The trajectory optimization is performed over a finite time $T_{h}$[61], where $T_{h}$ is chosen to be long enough to enable the trajectory plan to cover the entire motion from the robot’s initial position to the goal. The duration of a trajectory plan is determined by the sum of the control input durations; so, to enforce a finite time horizon, we introduce a constraint $\sum_{k=0}^{N-1}t_{k}=T_{h}$. The resulting classical trajectory planning problem formulation is given in Equation (3).    

(3) $$\begin{array}{cc} \min_{u_{0}\ldots u_{N-1}}\; & \sum_{k=0}^{N-1}\left|\right|x_{g}{\left|\right|}^{2}\\ \mathrm{subject}\mathrm{to}:\; & \forall k\in \left\{0,1\ldots N-1\right\},0\leq v_{k}\leq v_{max}\\ \; & \forall k\in \left\{0,1\ldots N-1\right\},-a_{max}\leq a_{k}\leq a_{max}\\ \; & \sum_{k=0}^{N-1}t_{k}=T_{h} \end{array}$$

Solving this optimization problem would produce triangular or trapezoidal velocity profiles depending on the distance to be traveled. In our motion corpus, velocity profiles that use the saccade and increment variants, or hesitation and pause motion sequences, are not purely trapezoidal or triangular and cannot be generated using this approach since they do not represent the optimal trajectory, e.g., hesitations introduce a deceleration in the middle of the motion, which increases the time taken to arrive at the goal position. The acceleration and maximal velocity values may also be different when compared to those associated with the three kinematics types.

In order to shape the trajectories produced by the optimization, we propose designing novel constraints that extend this optimization problem to restrict the set of valid control sequences based on the values of our prosody motion corpus variables.

### 4.4. Prosody Constraint Formalization

In this section, we propose novel constraints that model each of the motion corpus prosody parameters such that a trajectory whose motion phases satisfy the constraint is representative of the corresponding motion corpus parameter value.

#### 4.4.1. Integration of Motion Sequences

The corpus defined six motion sequences denoted A through F. We do not consider sequences E and F in our trajectory generation since they are simply truncated versions of sequence A. The four remaining sequences represent the possible combinations of two concepts: pauses and hesitations. Pauses are used in sequences A and C, and hesitations are used in sequences C and D.

**Figure 7:**
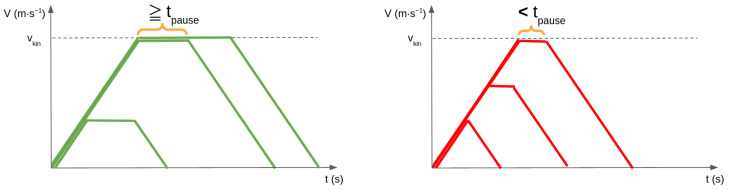
Illustration of the pause constraint. **Left**: valid trajectories. **Right**: invalid trajectories due to insufficient length of the constant velocity phase.

Trajectories using pause Motion Sequences (i.e., sequences A or C) require that an acceleration or deceleration phase $a_{k-1}\neq 0$ is followed by a constant velocity phase $a_{k}=0$ with a duration $t_{k}$ greater or equal to the pause length $t_{pause}=300$ ms (see Figure 7). This constraint is expressed in Equation (4), in such a way that it describes what should not occur: if the previous phase is not a constant velocity phase, the current phase is not the same acceleration as the previous, and the current phase is not a constant velocity phase as long or longer than a pause, then this trajectory does not satisfy the pause constraint.

(4) $$\begin{array}{cc} PauseConstraint(U)↔ & \forall k\in \left[1,N-1\right],\\ \; & \neg \left(a_{k-1}\neq 0\wedge a_{k}\neq a_{k-1}\wedge \neg \left(a_{k}=0\wedge t_{k}\geq t_{pause}\right)\right) \end{array}$$

Trajectories using hesitation motion sequences (i.e., sequences C or D) incorporate a deceleration from the current velocity down to some lower velocity, followed by the opposite acceleration, both with duration $t_{h}$. This hesitation deceleration should occur immediately after the end of an acceleration phase and then at regular time intervals $t_{h\_interval}$. Hesitation deceleration phases are enforced by the constraint in Equation (5). The variable $t_{since\_hesit}$ is introduced to ensure that a hesitation deceleration is added after $t_{h\_interval}$. Hesitation accelerations are enforced by the constraint in Equation (6). The variable $type_{k}\in \left\{normal,hesitation\right\}$ is introduced so that a hesitation acceleration is not enforced after a normal deceleration. These constraints are combined to enforce the hesitation motion sequence (Equation (7)).

(5) $$\begin{array}{cc} HesitationDeceleration(U)↔ & \forall k\in \left[1,N-1\right],\\ \; & \left(a_{k-1}=a_{kin}\vee t_{since\_hesit}\geq t_{h\_interval}\right)\\ \; & \wedge \neg (a_{k}=-a_{kin}\wedge t_{k}=t_{h}) \end{array}$$

(6) $$\begin{array}{cc} HesitationAcceleration(U)↔ & \forall k\in \left[1,N-1\right],\\ \; & \left(a_{k-1}=-a_{kin}\wedge type_{k-1}=hesitation\right)\\ \; & \wedge \neg (a_{k}=a_{kin}\wedge t_{k}=t_{h}) \end{array}$$

(7) $$\begin{array}{cc} HesitationConstraint(U)↔ & \neg HesitationDeceleration(U)\\ \; & \wedge \neg HesitationAcceleration(U) \end{array}$$

#### 4.4.2. Integration of Variants

A trajectory using the smooth variant should result in acceleration and deceleration phases longer than a given minimal duration $t_{smooth}$, such that the trajectory does not resemble the saccade variant. We simply implement a lower bound constraint on the length of motion phases $t_{smooth}=300$ ms (Equation (8)). By applying this definition of the smooth variant, we are also limiting the robot’s ability to perform short motions, which would require an acceleration and deceleration with shorter phase lengths. If instead we decide that such short motions should be considered valid smooth motions, the constraints could be modified to allow short two-phase trajectories if they start and end at zero velocity.

(8) $$\begin{array}{cc} SmoothConstraint(U)↔ & \forall k\in \left[0,N-1\right],t_{k}>=t_{smooth} \end{array}$$

The increment variant requires acceleration phases to be split into increments such that the robot performs a constant velocity phase of duration $t_{pause}=300$ ms when reaching certain velocities, which are multiples of $v_{increment}=\frac{1}{3}stoppingTime\left(v_{kin},a_{kin}\right)$. The first part of the constraint (Equation (9)) enforces that all acceleration or deceleration phases should end at one of the increment velocities. The second part of the constraint enforces that all acceleration and deceleration phases must be followed either by a pause phase or by their opposite phase (Equation (10)), i.e., an acceleration or deceleration phase cannot be extended, since it would violate the first constraint. The increment constraint is expressed by combining these two conditions in Equation (11).

(9) $$\begin{array}{cc} ValidVelocity(U)↔ & \forall k\in \left[0,N-1\right],\\ \; & v_{k}=i\times v_{increment},i\in N \end{array}$$

(10) $$\begin{array}{cc} BreakAccelerationPhase(U)↔ & \forall k\in \left[1,N-1\right],\\ \; & a_{k-1}\neq 0\wedge \left(\left(a_{k}=0\wedge t_{k}=t_{pause}\right)\vee a_{k}=-a_{k-1}\right) \end{array}$$

(11) $$\begin{array}{cc} IncrementConstraint(U)↔ & ValidVelocity(U)\\ \; & \wedge BreakAccelerationPhase(U) \end{array}$$

The saccade variant differs from the other prosody variables, since we do not formalize it as a constraint in the optimization problem, but rather as a post-processing step. In our motion corpus, saccades correspond to oscillations of the velocity over time, with a high frequency and low amplitude, since the aim of this variant is to reproduce stuttering or shaking. The saccade variant can, therefore, be accomplished by adding a time-varying offset given by a triangular wave to the profile given by planning under the smooth variant constraint, without rendering the trajectory invalid. We use a period π=0.02 s and an amplitude dependent on the kinematics type: $A\_low=0.02\;m\;\cdot \;s^{-2}$, $A\_medium=0.05\;m\;\cdot \;s^{-2}$, and $A\_high=0.07\;m\;\cdot \;s^{-2}$.

#### 4.4.3. Integration of Kinematics Types

The kinematics type specifies an acceleration value or, in other words, the slope of the velocity profile in acceleration and deceleration phases. When a kinematics type is specified, the robot must accelerate using that specific value, which we enforce with a constraint on the space of control inputs $u_{k}$ of the robot. The acceleration values $a_{k}$ are constrained to the finite set $\left\{-a_{kin},0,a_{kin}\right\}$. The value of $a_{kin}$ is determined by the kinematics type (high, medium, or low).

(12) $$\begin{array}{cc} KinematicsAcceleration(U)↔ & \forall k\in \left[0,N-1\right],\\ \; & a_{k}\in \left\{-a_{kin},0,a_{kin}\right\} \end{array}$$

The kinematics type specifies a maximum velocity that the robot should not exceed, which we enforce with an inequality constraint $v_{k}\leq v_{kin}$. The kinematics type also captures the amount of energy used for a motion; hence, the velocity should approach $v_{kin}$ when possible. For example, accelerating to $v_{k}<v_{kin}$, performing a constant velocity phase, and decelerating should not occur. The constraint in Equation (13) forces constant velocity phases to only be planned at the maximum velocity. The velocity and acceleration constraints are combined to form the overall kinematics constraint expressed in Equation (13).

(13) $$\begin{array}{cc} KinematicsVelocity(U)↔ & \forall k\in \left[0,N-1\right]\\ \; & 0\leq v_{k}\leq v_{kin}\wedge \neg \left(a_{k}=0\wedge v_{k}\notin \left\{v_{kin},0\right\}\right) \end{array}$$

(14) $$\begin{array}{cc} KinematicsConstraint(U)↔ & KinematicsAcceleration(U)\\ \; & \wedge KinematicsVelocity(U) \end{array}$$

### 4.5. Trajectory Planning and Open-Loop Control

Our set of proposed prosody constraints $PC_{offline}$ is summarized in Table 4. These constraints are integrated into our new problem formulation given in Equation (15), which retains the same control variables and cost function as the previous formulation. Each constraint enforces trajectory properties that are specific to a given corpus variable value. In order for the trajectory planning to produce plans that reflect the desired movement prosody, we must select a subset $PC_{active}$ of the constraints from $PC_{offline}$. For example, in order to plan trajectories according to the corpus variable values of pause motion sequence, high kinematics, and smooth variant, we define the subset $PC_{active}=\left\{Pause,Kinematics,Smooth\right\}$, and set $a_{kin}=a_{high}$ and $v_{kin}=v_{high}$ to specify which kinematics type should be applied.

(15) $$\begin{array}{cc} \min_{u_{0}\ldots u_{N-1}}\; & \sum_{k=0}^{N-1}\left|\right|x_{g}{\left|\right|}^{2}\\ \mathrm{subject}\mathrm{to}:\; & \left\{\begin{array}{c} \;PC_{active}\subset PC_{offline}\;,\\ \;\sum_{k=0}^{N-1}t_{k}=T_{h}\;. \end{array}\right. \end{array}$$

#### 4.5.1. Trajectory Planning

In order to solve the optimization problem (15), given that both optimization variables $a_{k}$ and $t_{k}$ are discretized, we use a tree-based approach to search the space of trajectories with a fixed length of *N* motion phases. We approach the problem as building a tree of possible trajectories starting from the robot’s current state, iteratively adding phases in a depth-first fashion. A node corresponds to a state $x_{k}$, an edge corresponds to a motion phase $u_{k}$, and a path of depth *N* corresponds to a trajectory. The root node corresponds to the robot’s initial state. The set of possible control inputs for the *k*th phase is given as $u_{k}\in A\times T$, where $A=\{a_{kin},0,-a_{kin}\}$ is the set of acceleration values determined by the kinematics type, and $T=\{dt,2dt,\ldots t_{max}\}$ is the set of possible phase durations. The maximum phase duration $t_{max}$ is computed by subtracting the durations of previous phases and the minimum duration of the following phases from the planning horizon duration $T_{h}$ (Equation (16)). The discretization level for the accelerations is already enforced by the kinematics constraint. For the durations, we choose a discretization dt=100 ms, which is short enough to enable all prosody constraints to be enforced accurately (such as the 300 ms pause constraint) and also long enough to maintain a low number of possible trajectories.

(16) $$\begin{array}{c} t_{max}=T_{h}-\sum_{i=0}^{k}t_{i}-\left(N-k\right)dt \end{array}$$

Pseudo-code for our algorithm is given in Algorithm 1. In order to expand the tree, we select a control $u_{k}\in A\times T$ (line 5) and compute the state $x_{k+1}$ that would result from executing $u_{k}$ (ForwardSimulation function, line 6). We then verify whether this extension of the trajectory satisfies the constraints using the CheckConstraints function (line 7). This function evaluates each constraint in problem (15), returning a Boolean value indicating whether the edge corresponding to control $u_{k}$ is valid. If adding the edge to the tree causes the corresponding trajectory to violate any of the constraints, the edge is discarded. If the edge complies with the constraints, we add the node corresponding to the state $x_{k+1}$ to the tree (lines 8–9). This process is repeated for all controls $u_{k}$, after which we select the next node from which to expand the tree in a depth-first fashion (line 10).

The result is a tree of depth *N*, where each leaf node represents the last state of a fully prosody-compliant trajectory. The EvaluateTrajectories function exhaustively evaluates the trajectories according to the cost function from problem (15). The minimum-cost sequence of control inputs $U^{*}$ is then used as the input to the open-loop control algorithm described in the next paragraph, which executes the control inputs with appropriate timing.
**Algorithm 1:** Prosody-aware trajectory planning **Input**:$x_{g}$, goal point. $x_{\mathrm{init}}$, initial state. $PC_{active}\subset PC_{offline}$, set of active prosody constraints. A, set of phase accelerations. T, set of phase durations. *N*, number of motion phases.**Output**: $U^{*}=\left\{u_{0},u_{1}\cdots ,u_{N-1}\right\}$, phases of the optimal trajectory.**Notations:**$x_{k}=\left[x_{k},v_{k}\right]$, *k*th robot state. $u_{k}=\left[a_{k},t_{k}\right]$, *k*th motion phase. *T* trajectory tree.**Algorithm:**
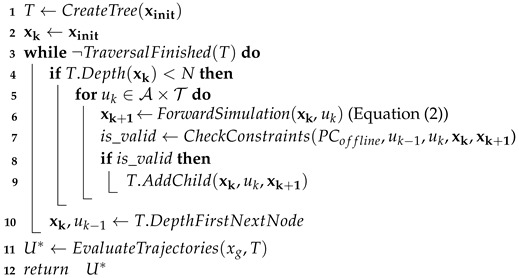


#### 4.5.2. Open-Loop Control

Algorithm 2 describes the overall process to execute a prosody compliant trajectory in an open loop fashion. It uses planning Algorithm 1 as a subroutine. The input to the control algorithm is the goal position $x_{g}$ given in the robot’s local coordinate frame as well as a selection of prosody constraints $PC_{active}$. We plan the trajectory using Algorithm 1 to solve the optimization problem given in Equation (15), obtaining the optimal trajectory $U^{*}$. We then simply iterate over the controls $\left\{u_{0},u_{1}\ldots u_{N-1}\right\}$, sending the corresponding acceleration command $a_{t}$ to the motors and waiting for the duration $t_{t}$ of the motion phase to elapse before sending the next command.
**Algorithm 2:** Open-loop control**Input**: $x_{g}$, goal point. $PC_{offline}$, set of prosody constraints.**Output**: $a_{t}$, acceleration command sent to the motors.**Notations:**$x_{0}=\left[x,v\right]$, initial state of the robot. $u_{t}=\left[a_{t},t_{t}\right]$, motion phase executed at time *t*. $U^{*}=\left\{u_{0},u_{1}\cdots ,u_{N-1}\right\}$, sequence of motion phases describing the trajectory.**Algorithm:**
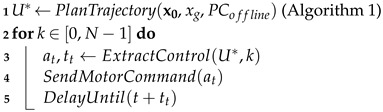


## 5. Implementation and Validation

### 5.1. Implementation

**Figure 8:**
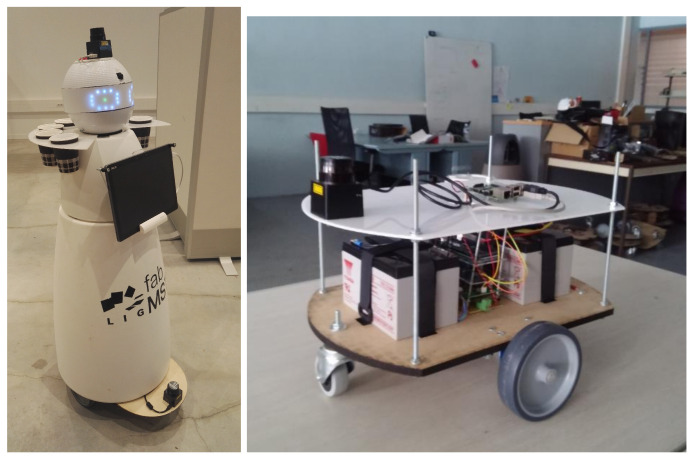
**Left**: RobAIR mobile robot. **Right**: RobAIR base.

Firstly, we present the RobAIR wheeled mobile robot platform shown in Figure 8. The RobAIR platform [62] is developed by the FabMASTIC fab lab at the Université Grenoble Alpes, where it serves both as a platform for teaching robotics, student projects, as well as for research. The robot is 1.20 m high, and has a diameter of 0.50 m at its widest point—at the base. The robot has a differential drive configuration, can reach a maximum velocity of $0.8\;m\;\cdot \;s^{-1}$, and accelerates at $2.667\;m\;\cdot \;s^{-2}$. Two Hokuyo URG-04-LX-UG01 laser range-finders are mounted on the robot’s head and at the base in order to detect obstacles and track people while navigating.

Certain parameters of our algorithm must be selected based on the types of movement prosody the robot should produce. We employed N=10 motion phases in order to be able to plan the most complex motions such as those using the increment variant. The time discretization was dt=100 ms in order to maintain a fine temporal resolution so that constraints such as the 300 ms pause may be accurately enforced. The short time discretization also allows for more accurate position tracking. When deploying our algorithm on a given robot, the velocity and acceleration constraint values should be selected such that they are within the specifications of the robot motor hardware to ensure that the generated trajectories are feasible.

**Figure 9:**
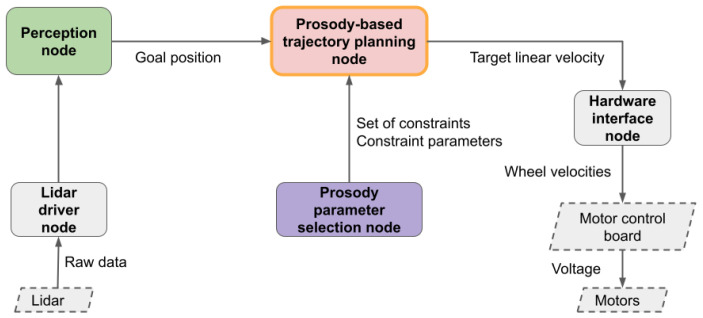
High-level architecture of our system. ROS nodes are represented with rounded boxes; hardware devices are represented with dashed boxes.

Algorithm 2 is implemented as an ROS node in C++. One planning cycle takes 50 ms on average and no more than 100 ms on a single core of a low-power tablet PC (Intel i5−8365U). Figure 9 shows the overall architecture. The goal point $x_{g}$ to be reached is given by a LIDAR-based perception module, allowing the robot to be driven to a person detected by a multiple hypothesis tracker based on clustering of the laser data. The planning node then uses Algorithm 1 to plan the trajectory to $x_{g}$. The planned acceleration commands are converted to sequences of linear velocity commands, given that our motors do not allow acceleration-based control. The planner node sends these velocity commands at 10 Hz to the hardware interface node, ensuring accurate timing. The linear velocity commands are finally translated into wheel velocities and sent to the motors. The set of constraints $PC_{active}$ used by the planner can be altered by the means of a prosody parameter selection node, which implements a simple ROS Dynamic Reconfigure interface to save and load parameter presets to represent the different movement prosody styles.

### 5.2. Validation

In this section, we demonstrate the ability of our planning algorithm to produce trajectories that accurately reproduce the different types of movement prosody defined by the combination of corpus variables while ensuring the robot reaches its goal. Plots of the velocity commands from our planner show that they are stable and consistent with the desired prosody. We also plot the raw encoder-based velocity estimation, showing that the commanded velocities are indeed achievable by our robot platform, thanks to our planner and prosody constraints taking the robot’s mechanical limits into account. Unless stated otherwise, the prosody used in these examples are the medium kinematics, smooth variant, no pauses, and no hesitations.

#### 5.2.1. Point-to-Point Trajectory Execution

**Figure 10:**
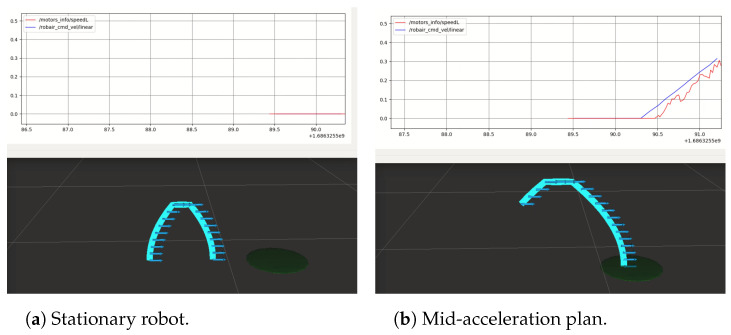

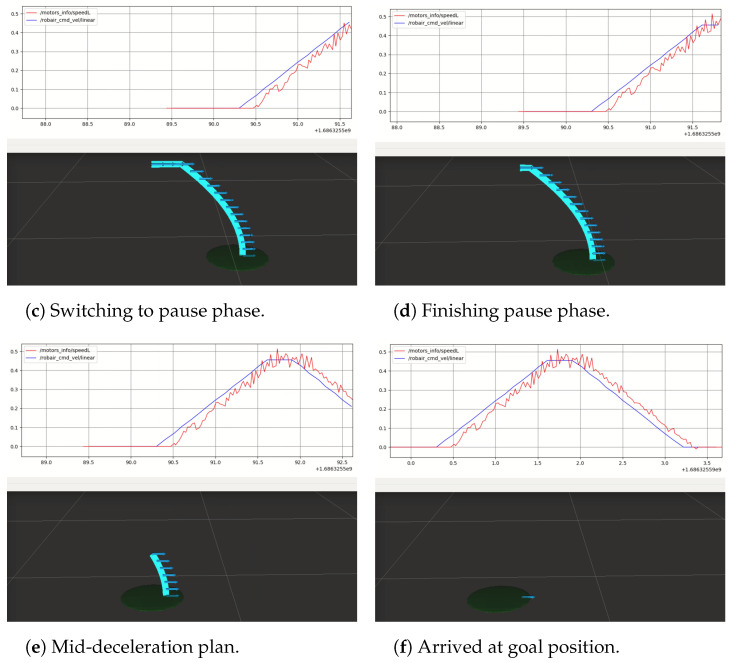
**Top**: past command velocities issued at 10 Hz (blue) and encoder-based odometry estimated at 40 Hz (red) in $\;m\;\cdot \;s^{-1}$, plotted with respect to time (s). **Bottom**: visualization of the planned trajectory’s velocity, discretized into time intervals of length dt=100 ms. The robot stops within 10 cm of its goal position (green).

Firstly, we demonstrate the ability of our proposed trajectory planning and control algorithms to successfully drive the robot towards a goal position. Figure 10 shows the execution of a plan consisting of an acceleration, constant velocity, and deceleration that have been optimized to reach the goal while satisfying all movement prosody constraints on the acceleration, velocity, and timing of the motion. In this case, the constraints involved are the acceleration, maximum velocity, as well as the pause constraint, requiring the robot to perform a 300 ms constant velocity phase before decelerating. In the remainder of this section, we focus on demonstrating the accurate reproduction of the movement prosody features in the planned velocity profiles.

#### 5.2.2. Kinematics

The three kinematics types (low, medium, and high) require different accelerations, and different maximal velocities. We show examples of motions produced by running our planner with each of the kinematics types. We plot the raw odometry estimate of velocity based on the integration of the motor’s encoder readings over time in order to demonstrate how the physical robot platform responds to the velocity commands. The unfiltered odometry is noisy due to the cheap encoder sensors, whereas the true motion of the robot is smooth. The plots of the unfiltered commands allow us to see that the response time of the motors is very fast, allowing the robot to accurately track even the most subtle and fast changes in the velocity commands.

**Figure 11:**
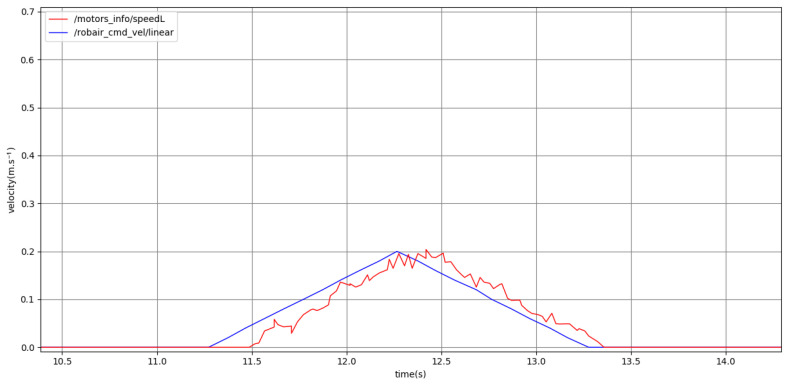
Plot representing the full point-to-point motion to a goal point, using low kinematics. Past command velocities shown in blue and unfiltered odometry shown in red, both given in $\;m\;\cdot \;s^{-1}$.

Figure 11 shows a short motion with the low kinematics. The goal point is close enough that the robot only accelerates to $0.20\;m\;\cdot \;s^{-1}$, slightly below the low kinematics maximum of $0.24\;m\;\cdot \;s^{-1}$. The slope of the commanded velocity profile corresponds to the low kinematics acceleration of $0.2\;m\;\cdot \;s^{-2}$ as expected, and the estimated velocity also follows the commanded velocity closely.

**Figure 12:**
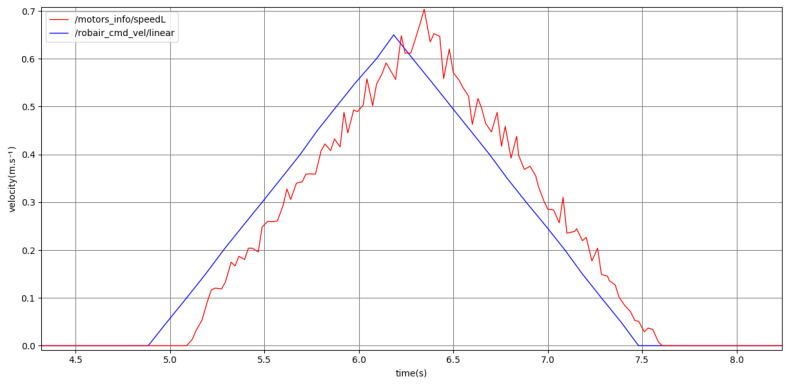
Plot representing the full point-to-point motion to a goal point using high kinematics. Past command velocities shown in blue and unfiltered odometry shown in red, both given in $\;m\;\cdot \;s^{-1}$.

Figure 12 shows a short motion with high kinematics. Again, the goal point is close enough such that the robot does not need to accelerate to the maximum high kinematics velocity of $0.72\;m\;\cdot \;s^{-1}$. The robot accelerates to $0.65\;m\;\cdot \;s^{-1}$, with an acceleration of $0.5\;m\;\cdot \;s^{-2}$, clearly distinguishing the motion from the low kinematics setting. The profiles shown in the following subsections all use the medium kinematics setting, which is also distinct from the low and high settings.

#### 5.2.3. Pause and Hesitation Sequences

**Figure 13:**
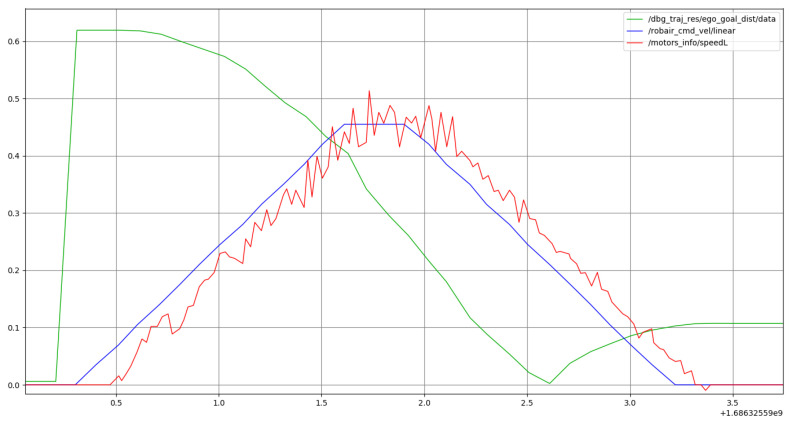
Plot representing the full point-to-point motion to a goal point. Past command velocities shown in blue and unfiltered odometry shown in red, both given in $\;m\;\cdot \;s^{-1}$. Distance to the goal in m shown in green.

Figure 13 shows the plot of the robot’s velocity, and distance during a point-to-point motion to a goal placed 62 cm from the robot without obstacles. The active prosody constraints are the medium kinematics type, smooth variant, and pauses. The plans generated by the controller result in a velocity profile that conforms to the prosody constraints—a linear acceleration and deceleration phase, separated by a pause phase of 300 ms—and drives the robot towards the goal point (video with visualization of the plan execution using Rviz (shown at 0.2× speed for clarity): https://cloud.univ-grenoble-alpes.fr/s/f5G8kQR4rMx6MWi (accessed on 25 March 2024)).

**Figure 14:**
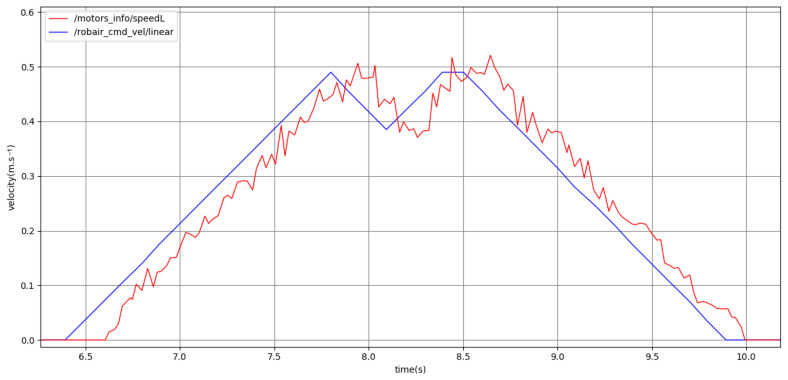
Point -to-point motion using the hesitation sequence (without pauses) and medium kinematics. Past command velocities shown in blue and unfiltered odometry shown in red, both given in $\;m\;\cdot \;s^{-1}$.

Figure 14 shows the plot of the robot’s velocity when using the hesitation constraint. Once again, the generated plan for the robot’s velocity enforces the hesitation feature by including the succession of a deceleration and acceleration upon reaching the robot’s maximum velocity. The robot is able to accurately reproduce motions including the hesitation feature.

#### 5.2.4. Increment and Saccade Variants

**Figure 15:**
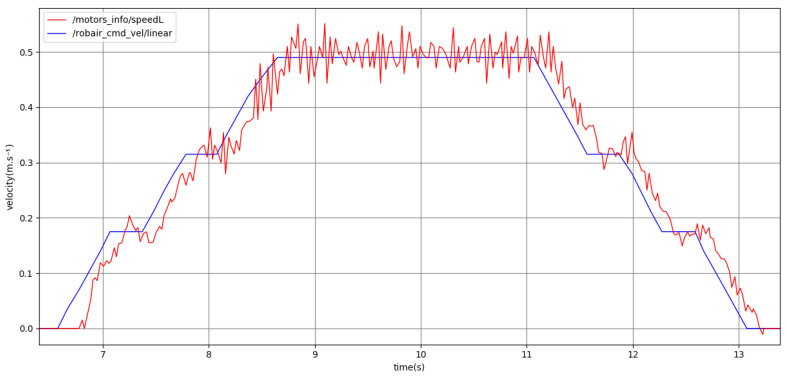
Point -to-point motion using the increment variant and medium kinematics. Past command velocities shown in blue and unfiltered odometry shown in red, both given in $\;m\;\cdot \;s^{-1}$.

In this subsection, we demonstrate motions planned under the increment or saccade variant constraints. Figure 15 shows a long increment motion, allowing the robot to reach the maximum velocity for the medium kinematics type. The planner correctly inserts constant velocity phases at regular intervals, which are tracked by the robot motors, reproducing the stepped acceleration pattern.

**Figure 16:**
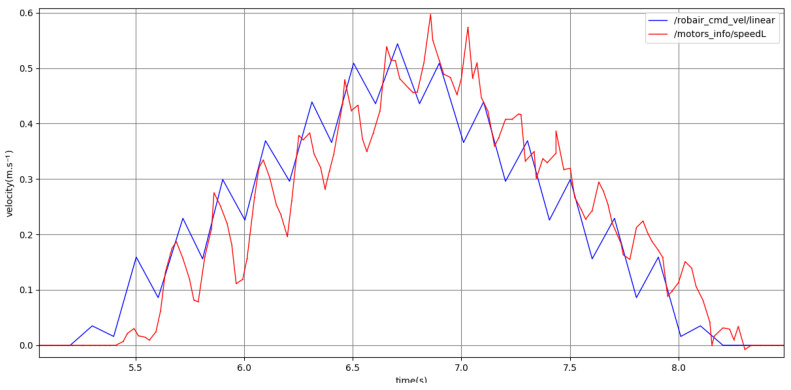
Point -to-point motion using the saccade variant and medium kinematics. Past command velocities shown in blue and unfiltered odometry shown in red, both given in $\;m\;\cdot \;s^{-1}$.

Figure 16 shows a short saccade motion without pauses. The planner reproduces the expected oscillation in the velocity commands, and the robot is able to accurately track the rapid changes in the requested velocity, enabling the robot to perform the stuttering, saccadic movements as desired.

## 6. Discussion

### 6.1. Generalization of the Human Perception Model

Our model of human perception was derived from the analysis of experimental data from our online study and in-person studies presented in our prior work [30]. The results show that accelerations, velocities, and timing have significant impacts on the social perception of our mobile robot. However, prior studies have shown that the size of a robot [63], its shape and color [64], as well as its human-like or machine-like appearance [65] may also impact interaction. While our study did include three appearance variables (head orientation, eye shape, and base stability), further studies are necessary to explore the generalization of our model to different robot types.

Our experiments were designed in such a way that the robot was not shown in any specific scenario or social environment, since prior research has shown that a robot’s behavior may be perceived differently and lead to different acceptance outcomes in different social settings, such as two different hospital services in [16]. Further work is also necessary to study how the robot’s task, its social role, and its social environment may impact and alter human’s social perceptions of the robot.

### 6.2. Limitations of the Trajectory Planning Algorithm

The main limitation of our algorithm is that it assumes static and known environments. In many real-world use cases, the environment will be dynamic and the robot’s perception of pedestrians or other dynamic obstacles will be uncertain. One approach is to use our algorithm as a global planner and, subsequently, attempt to follow the global plan and avoid obstacles when necessary; however, such an approach would not guarantee that the robot’s movement style is accurately maintained by the obstacle avoidance algorithm. Another approach to adapt trajectory planning algorithms to deal with changing and uncertain environments is to perform frequent re-planning [61]. However, given the subtle and time-dependent nature of the motion features we aim to reproduce, this would lead to inconsistencies in the robot’s motion without careful consideration of the re-planning mechanism in the algorithm design, hence changing the human’s social perception of the robot. Extending our algorithm to dynamic environments while maintaining accurate control over the robot’s motion features is therefore non-trivial and requires further research.

### 6.3. Ethical Considerations

The statistical analysis of our perception experiment showed that the mobile robot’s motion features could significantly impact a human’s social perception of the robot. Subsequently, we proposed an approach to formalize the relevant motion features and integrate them into an optimization-based trajectory planner, taking a first step towards controlling the motion features responsible for altering social perceptions. On the one hand, these contributions may be used to analyze existing navigation algorithms to understand their impact on people and potentially avoid generating inappropriate social attitudes. On the other hand, altering the social perceptions of humans must be conducted with care and while considering the goal of such manipulations. For example, generating an impression of frailty can lead to a person being more engaged and active in an interaction, which may be useful in assistive or care use-cases; however, this may also induce attachment effects, which are not well understood [26,66]. Determining when and how to alter the generated social attitude in a given deployment scenario should be determined with the input of domain experts and end-users in addition to HRI researchers.

## 7. Conclusions and Future Work

In this paper, we studied how changes in a mobile robot’s motion features alter human social perception of the robot, in order to better integrate robots into human environments. The statistical analysis of a perception experiment with n=100 participants showed that motion features such as the robot’s acceleration, velocity, and saccades have statistically significant impacts on human perception of social attitudes in mobile robots. Each of these features altered the probability of perceiving the robot as aggressive or gentle, authoritative or polite, or sturdy or frail by up to 30 percentage points. These results demonstrate that even subtle motion features have strong impacts on social perception, and therefore on the acceptance and integration of robots in human environments. Subsequently, we proposed a trajectory planning algorithm that can be configured to integrate these motion features into the trajectory while performing a point-to-point navigation task. We formulated the problem as a constrained optimization and derived a novel set of constraints to enforce the motion features that impact human social perception of the robot. The algorithm was implemented and validated on a real mobile robot, demonstrating that the trajectories produced by our planner accurately reproduce the features used in our perception experiment. Our algorithm enables a mobile robot’s motion to be adjusted according to the desired social perception of the robot by humans, which was previously not possible using existing social navigation algorithms. Providing explicit control over how the robot is perceived ensures that the robot’s actions are appropriate with respect to its role and the people it is interacting with.

In future work, we aim to extend our algorithm to handle dynamic uncertain environments by introducing temporal coherence constraints to enable accurate re-planning. We also plan to deploy our algorithm in a realistic task to evaluate the impact of the different trajectory styles on humans when the interaction is situated in a social environment (preliminary video of a participant interacting with our robot using the proposed algorithm, configured to convey a confident attitude: https://cloud.univ-grenoble-alpes.fr/s/GdnDKQbKD9GEgnG (accessed on 25 March 2024)). Further experiments should also be conducted in different social environments and scenarios, as well as with different robot types, to determine the extent to which the model of human perception generalizes.

## Figures and Tables

**Table 1:** Kinematics type parameters.

Parameter	Low	Medium	High
*a*	$0.2\;m\;\cdot \;s^{-2}$	$0.35\;m\;\cdot \;s^{-2}$	$0.5\;m\;\cdot \;s^{-2}$
$v_{min}$	$0.05\;m\;\cdot \;s^{-1}$	$0.15\;m\;\cdot \;s^{-1}$	$0.25\;m\;\cdot \;s^{-1}$
$v_{max}$	$0.25\;m\;\cdot \;s^{-1}$	$0.50\;m\;\cdot \;s^{-1}$	$0.75\;m\;\cdot \;s^{-1}$
0 to $v_{max}$	1.25s	1.42s	1.5s
$v_{min}$ to $v_{max}$	1.0s	1.0s	1.0s

**Table 2:** Perceptual scales.

Adjective 1	Adjective 2
Aggressive	Gentle
Authoritative	Polite
Seems Confident	Doubtful, Hesitant
Inspires confidence	Doesn’t inspire confidence
Nice	Disagreeable
Sturdy	Frail
Strong	Weak
Smooth	Abrupt
Rigid	Supple
Tender	Insensitive

**Table 3:** Marginal effects of the corpus variables on the perceptual scales in percentage points (p.p.). * p<0.05, ** p<0.01, *** p<0.001.

−	Aggressive	Authoritative	Confident	Inspires Conf.	Nice
**+**	**Gentle**	**Polite**	**Hesitant**	**Does Not**	**Disagreeable**
Kin. high	−28 ***	−24 ***	−17 ***	7 ***	15 ***
Kin. low	24 ***	22 ***	15 ***	−8 ***	−15 ***
Kin. medium	4 ***	3 *	2	1	1
Sequence A	2	4	−3	−1	2
Sequence B	2	7 **	5 *	−1	−4
Sequence C	0	2	19 ***	9 ***	1
Sequence D	1	2	27 ***	13 ***	1
Sequence E	−6 *	−10 ***	−28 ***	−9 ***	3
Sequence F	1	−5	−21 ***	−11 ***	−3
Var. increment	8 ***	6 ***	−1	−4 **	−3 *
Var. saccade	−14 ***	−8 ***	22 ***	20 ***	10 ***
Var. smooth	6 ***	3	−21 ***	−16 ***	−6 ***
Eyes none	1	5 **	4	3 *	3 *
Eyes round	7 ***	5 **	−1	−7 ***	−11 ***
Eyes squint	−8 ***	−10 ***	−3	4 *	8 ***
Stable	3 *	−2	−11 ***	−6 ***	−2
Unstable	−3 *	2	11 ***	6 ***	2
**−**	**Sturdy**	**Strong**	**Smooth**	**Rigid**	**Tender**
**+**	**Frail**	**Weak**	**Abrupt**	**Supple**	**Insensitive**
Kin. high	−15 ***	−20 ***	13 ***	−9 ***	13 ***
Kin. low	12 ***	17 ***	−14 ***	12 ***	−13 ***
Kin. medium	3 *	3 *	1	−2 *	0
Sequence A	−3	−3	−4	2	−1
Sequence B	9 ***	7 ***	0	3	−6 *
Sequence C	10 ***	9 ***	4	1	2
Sequence D	20 ***	18 ***	7 **	−5 *	2
Sequence E	−22 ***	−19 ***	−2	−2	6 *
Sequence F	−14 ***	−12 ***	−5 *	1	−2
Var. increment	−4 *	−2	−4 ***	3 *	−4
Var. saccade	27 ***	20 ***	15 ***	−9 ***	6 ***
Var. smooth	−24 ***	−18 ***	−10 ***	6 ***	−3
Eyes none	3	4 *	1	−1	6 ***
Eyes round	1	1	−7 ***	4 *	−14 ***
Eyes squint	−3	−6 **	6 ***	−3	8 ***
Stable	−16 ***	−11 ***	−4 ***	0	0
Unstable	16 ***	11 ***	4 ***	0	0

**Table 4:** Constraints forming the set $PC_{offline}$ used for offline planning.

Constraint	Equation
Pause	(4)
Hesitation	(7)
Smooth	(8)
Increment	(11)
Kinematics	(14)

## Data Availability

The raw data supporting the conclusions of this article will be made available by the authors on request.
